# Research and Method of Roughness Prediction of a Curvilinear Surface after Titanium Alloy Turning

**DOI:** 10.3390/ma12030502

**Published:** 2019-02-06

**Authors:** Andrzej Matras, Wojciech Zębala, Magdalena Machno

**Affiliations:** Production Engineering Institute, Mechanical Faculty, Cracow University of Technology, 31-155 Kraków, Poland; zebala@mech.pk.edu.pl (W.Z.); magdalena.machno@mech.pk.edu.pl (M.M.)

**Keywords:** turning, surface roughness, curvilinear surface, titanium alloy

## Abstract

This paper deals with the optimization of process parameters (such as cutting speed and feed rate) to minimize surface roughness in the turning of a titanium alloy (Ti-6Al-4V) workpiece with spherical shape. In the first part of the article, based on the results analysis, a mathematical model is developed. It is shown that cutting speed has little effect on the surface roughness. The second part of the paper presents the application of the developed method to optimize cutting data such as feed rate in order to obtain the surface roughness parameters Ra and Rz of the curvilinear surface of the titanium alloy workpiece at acceptable and aligned, values regardless of the surface shape and its tilted angle. A case study verifies the correctness of the proposed method. The machining time was substantially shortened in comparison to the non-optimized cutting process.

## 1. Introduction

Nowadays, titanium and its alloys are widely used in various areas, such as the aerospace, medical and automotive industries, due to their excellent properties (e.g., high strength-to-weight ratio and good corrosion resistance, relatively low density, high-temperature properties, excellent creep, biocompatibility) [1]. Vanadium, molybdenum, manganese and aluminum are often alloying elements and provide high strength [2]. However, titanium alloys are classified as difficult-to-cut materials. The poor machinability of the materials is caused by their properties [3]. A rapid tool wear rate due to the low thermal conductivity and high chemical reactivity causes high cutting temperature at the cutting zone [4].

Difficult-to-cut materials generally make it challenging to obtain the required surface integrity, high performance and economic of machining [5]. The indicators depend mainly on the kind of machined workpiece and tool life [6]. Various methods and technologies have been developed to improve the quality of machined surface and to increase performance of machining [7]. Additionally, the decrease of manufacturing costs plays an important role. Necessary optimization should simultaneously provide a short machining time and obtain the required quality of surface roughness. To minimize costs and increase performance machining, application of different values of cutting data can be used.

Nowadays, to optimize machining process parameters, various evolutionary or meta-heuristic methods can be used, such as GA, PSO, ACO and ABC. The application of the techniques in optimizing machining process parameters has been proven in the literature [8]. Although these methods are applied in many practical cases, they characterize limitations related to their inherent search mechanism. The solutions of the techniques depend generally on the type of objective and constraint functions (linear, non-linear, etc.) and the type of variables used in the problem modeling (integer, binary, continuous, etc.). To predict the performance of machining processes, regression models based on experimental tests have been developed. These regression models can be solved by using traditional optimization methods which are sensitive to the initial assumption. Excellent solutions in the case of several input parameters is difficult [9].

In manufacturing technology, surface roughness is an important indicator that can affect the performance of mechanical parts (product wear, fatigue strength, tribological properties, corrosion resistance [10] and manufacturing cost [4]. Therefore, evaluating surface roughness parameters is significant. Hence, optimization is widely used to achieve the required surface quality and process performance. One of the numerous methods for surface roughness prediction and obtaining optimal cutting parameters is the Response Surface Methodology (RSM) [11].

In the turning process (one of the most used in manufacturing technology), the values of machining parameters (feed rate, cutting speed, and cutting depth), nose radius, cutting time, cutting fluid, and cutting forces are subjected to optimization [2,12]. The cutting data directly affects the surface roughness, dimensional accuracy, tool wear rate, machining performance and manufacturing costs. The selection of appropriate parameters in order to improve surface finish is difficult [13,14,15].

In the case of turning of titanium alloy, many researchers analyze the impact of machining parameters on surface roughness and optimize their values to provide the required surface quality. The authors [2] have developed mathematical models to predict the surface roughness after the turning process of titanium alloy. The impact of the cutting parameters and the kind of tool materials on the surface roughness were examined. The optimum conditions obtained for uncoated tools are cutting speed *v_c_* = 80 m/min, feed *f* = 0.05 mm/rev, and depth of cut *a_p_* = 0.25 mm. The authors proved that optimization techniques and mathematical models reduced the cost of machining. The proposed optimum process parameters resulted in an increase of surface finish. In [4], cutting parameters such as feed rate, cutting speed, and depth of cut were used to predict surface roughness in turning of aerospace titanium alloy (gr5). The proposed model studied the effect on surface roughness when varying the turning parameters using the surface plots. The analysis of results showed the feed rate to be the most influential parameter on the surface roughness. Yang at al. [11] present the prediction model to predict the surface roughness and cutting parameters in the turning process of TC11 titanium alloy. It also indicates that the feed rate is the most important parameter influencing surface roughness, followed by cutting speed. The cutting depth has minimal effect.

In the literature, different approaches of prediction surface roughness in machining have been presented. Asiltürk et al. [16] used the Taguchi experimental test to design optimized turning parameters and to obtain the lowest degree of surface roughness parameters (Ra and Rz). The results of the study showed that the most influential factors included the feed rate and the interaction between feed rate and cutting speed over the surface roughness. Makadia et al. [17] proposed developing the surface roughness prediction model of AISI 410 steel with the aid of a statistical method under various cutting conditions, such as cutting speed, feed rate, depth of cut and tool nose radius. The results analysis showed that the feed rate is the main influencing factor on the roughness, followed by the tool nose radius and the cutting speed. Depth of cut proved to be an insignificant parameter on the surface roughness. Yamane et al. [18] presented a method for quantitatively estimating the cutting stability and the machining system stability in the turning process. This method used the machined surface roughness profile to evaluate the position of the cutter in order to obtain the cutting edge transferability and the stability in the feed direction and in the depth-of-cut direction. Three samples with different roughness were estimated using the proposed evaluation method. The results showed that it is possible to quantitatively evaluate cutting instability based on adhesion or built-up edge, as well as the system instability resulting from vibration during machining. In [19], the authors presented a method of predicting roughness profile by further developing a methodology linking the theoretically developed models to the real machining process conditions, which are different for different processing systems (machines). This is significant, because researchers usually focus on finding prediction models for the Ra and Rz (Rt) parameters. In this approach, the statistical equality of sampling lengths in surface roughness measurement proves to be a major parameter that provides information about the condition of the process with respect to surface roughness formation. The authors demonstrated that the indicator of the roughness profile condition can be successfully implemented in roughness profile prediction. Özel and Karpat [20] focused on the development of models based on feedforward neural networks in accurately predicting both surface roughness and tool flank wear in finish dry hard turning. The neural network models were trained based on the experimental data of measured surface roughness and tool flank wear. The authors assumed that the neural network models provided better prediction capabilities because they were able to model more complex nonlinearities and interactions in comparison to linear and exponential regression models. In [21], a model was proposed based on ANN (Artificial Neural Network) to predict surface roughness (Ra) in terms of cutting parameters (such as feed rate, cutting speed, depth of cut) during hard turning of AISI H13 tool steel with minimal cutting fluid application. The authors showed that the ANN model could be applied successfully in fixing the cutting parameters to achieve desired surface finish and to maintain the surface finish within the tolerance limits during automated hard turning of AISI H13 steel with minimal fluid application.

Nowadays, machined parts have more and more complicated and nonlinear shapes. However, there is a lack of prediction models of curvilinear surface roughness in the turning process. There is a need to provide aligned values of roughness parameters on the whole surface after the turning process. The task is especially difficult when difficult-to-cut materials are machined. The prediction of surface roughness parameters values is a challenge that can result from the variable impact of cutting forces and change in the surface tilted angle.

In this study, a new optimized method is presented that involves the prediction of the curvilinear surface roughness. The proposed method was formulated based on the experimental research results. The created model also results in a short machining time and low manufacturing cost. In the first part of the study, the results of experimental tests of turning six curvilinear surfaces made of Ti6Al4V alloy are presented. Selection of the cutting parameters plays an important role in achieving high cutting performance and the required surface roughness. The experimental research was focused on the impact of cutting speed and feed rate on the quality of the spherical surface (values of roughness parameters Ra and Rz) for various surface tilted angles. The second part of the paper presents the optimization procedure for obtaining aligned surface roughness parameters for the example spherical profile of the part.

## 2. Materials and Methods

### 2.1. Characterization of the Analysed Problem

Machining a curvilinear surface is performed with using CNC machines that apply a cutting insert with an R shape and using interpolated axes. Cutting speed is maintained at a constant value by modifying the rotational spindle speed value, which depends on the diameter of the machined surface fragment. During the tool movement along the curvilinear surface, modifications of the angles between the cutting insert flank face and work surface occur.

**Figure 1:**
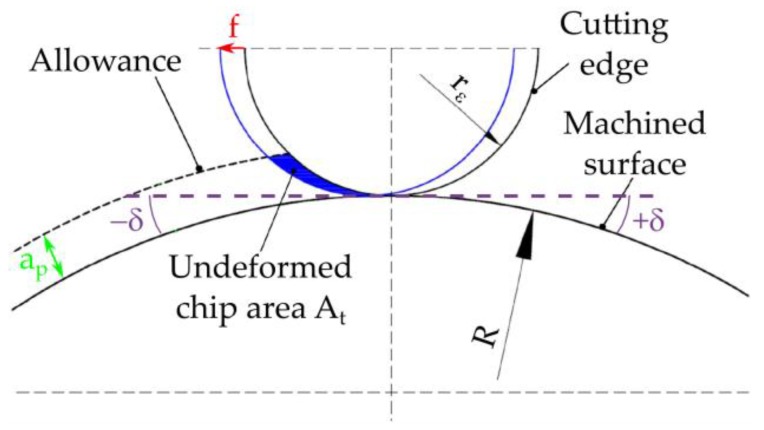
Scheme of forming undeformed chip area *A_t_*.

**Figure 2:**
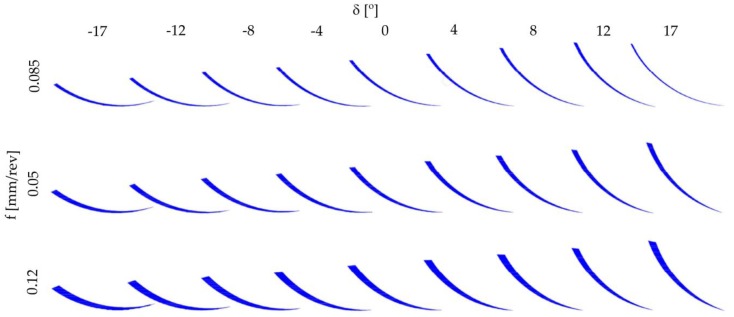
Shape changes of undeformed chip area *A_t_* for different feed rate *f* and surface tilted angle *δ*.

During the turning, the change of the undeformed chip area shape *A_t_* take place (Figure 1). The consequences of these phenomena are: machining with various parts of the cutting edge, change of the chip flow direction, and change of the machined surface roughness. Curvilinear surfaces with a radius of curvature *R* = 14 mm were subjected to turning with a cutting edge radius *r_ε_* = 1.588 mm. The selected parts of the undeformed chip areas *A_t_* for the different tilted angles of surface *δ* and feed rates *f* are shown in Figure 2. 

**Figure 3:**
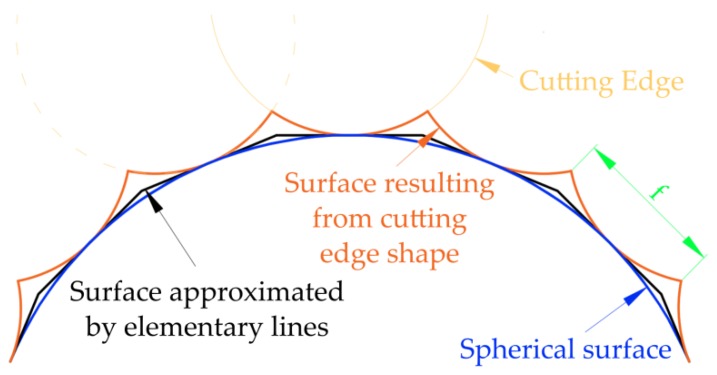
Scheme of determining the fundamental straight lines.

In further research, a simplification was applied. This was based on an approximation of a curved contour section part by means of elementary straight lines (Figure 3). The simplification enabled the assumption that the machining part was a tilted flat surface instead of a curvilinear surface. This simplification is often used for the analysis of the machining process of curved surfaces [22,23,24,25,26]. The assumption enables the reduction of the number of variables by excluding the impact of the curvature surface radius.

A literature analysis reveals a lack of studies and mathematical models observing the phenomenon. A similar phenomenon is observed during the milling of curvilinear surfaces using spherical cutters, which has been widely analyzed in the currently available scientific literature. The geometrical analysis confirms the insignificant effect of the simplification on the analyzed contour shape (e.g., for an arc with radius *R* = 14 mm, its bend deflection is 1.3 µm for 0.12 mm length) and the shape of the undeformed chip area. During the milling process, similar simplifications are applied for the analysis of the curvilinear surface.

### 2.2. Application of the Model for Surface Roughness Prediction

In the experimental study, the titanium alloy Ti6Al4V was used as a workpiece. The material was annealed to achieve an optimum combination of ductility, machinability, dimensional stability and structural stability in 750 °C temperature. The chemical composition and mechanical properties are shown in Table 1 and Table 2, respectively.

**Figure 4:**
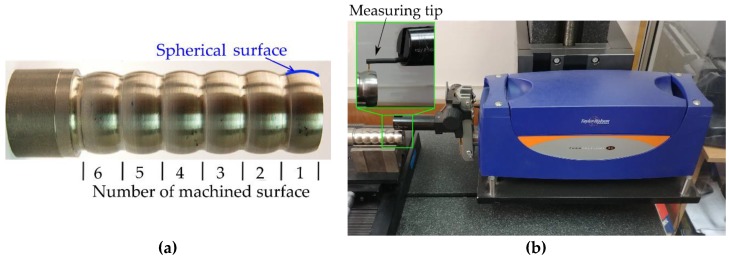
The workpiece with six machined spherical surfaces (**a**); and surfaces roughness measurement after turning (**b**).

During the experimental test, six spherical surfaces with radius of curvature *R* = 14 mm and length *L* = 10 mm were subjected to turning (Figure 4a) with a cutting edge radius *r_ε_* = 1.588 mm. Different initial parameters, such as feed rate *f* and cutting speed *v_c_*, were applied (Table 3). The tests were carried out based on a complete plan for 3 levels of feed rate and 9 levels of tilted angle. Additional experiments for 3 levels of cutting speed variation were made to determine its effect on the surface roughness. During the tests the tool holder with symbol RF123F10-2525B and sintered carbide cutting insert N123F1–0318–R0S05F were used. Turning of a curvilinear surfaces was performed using CNC machines that applied a cutting insert with an R shape and using interpolated axes. Cutting speed was maintained at a constant value by modifying the rotational spindle speed value, which depends on the diameter of the machined surface fragment. During the tool movement along the curvilinear surface, the modifications of the angles between the cutting insert flank face and work surface occur.

**Figure 5:**
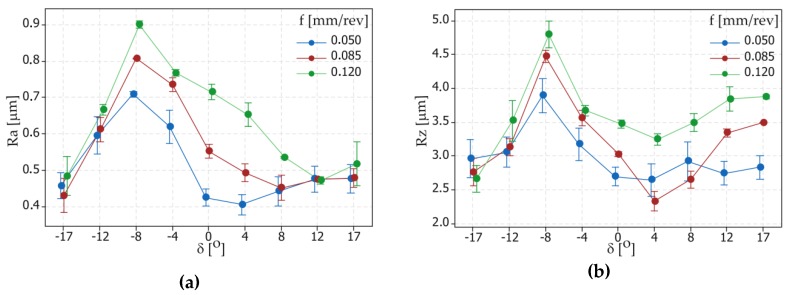
Graphs representing the relation between the tilted angle *δ* and surface roughness parameters: (**a**) Ra and (**b**) Rz; for different feed rate *f* values.

**Figure 6:**
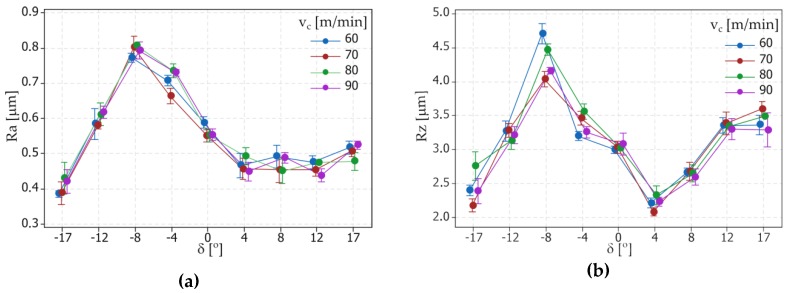
Graphs representing the relation between the tilted angle *δ* and surface roughness parameters: (**a**) Ra and (**b**) Rz; for different cutting speed *v_c_* values.

The surface topography was measured by using the Talysurf Intra 50 profilometer produced by the Taylor Hobson company (Leicester, UK) (Figure 4b). The fragments of the inclined surfaces at angles *δ* such as ±17°, ±14°, ±8°, ±4°, 0° were examined and used to measure surface roughness parameters such as Ra and Rz. To perform the surface roughness measurements, a measuring tip with a rounding radius of 2 μm was used. The measurements were made in the transverse direction to the machining marks (parallel to the measuring axis of the sample). A measurement speed of 1 mm/s was used. For measurements in the 2D system, the resolution in the X axis was equal to 1 μm, and five elementary sections 0.8 mm in length were applied. For measurements in the 3D system, an area of 0.8 × 0.8 mm was analyzed. The resolution in the X axis was 1 μm and in the Y axis was 10 μm. The research was carried out based on the ISO 4287 (for 2D measurements) and ISO25178 (for 3D measurements) standards with filter values such as λ_c_ = 0.8 mm and λ_c_ = 2.5 μm. The impact of different tilted angles of the surface (turning with different values of the feed rate *f* and cutting speed *v_c_*) on the surface roughness is presented in Figure 5 and Figure 6, respectively.

**Figure 7:**
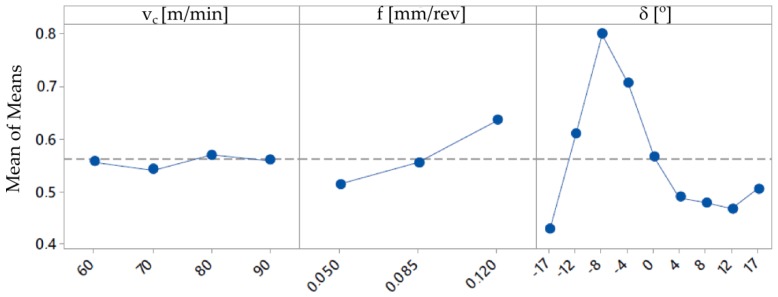
Graphs representing the relation between the surface roughness parameter Ra and the cutting speed *v_c_*, feed rate *f* and tilted angle *δ*.

**Figure 8:**
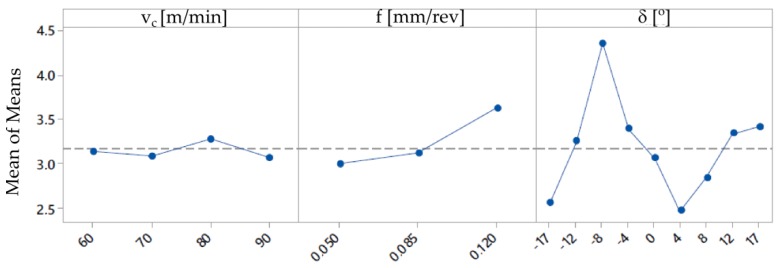
Graphs representing the relation between the surface roughness Rz and the cutting speed *v_c_*, feed rate *f* and tilted angle *δ*.

On the basis of the obtained measurement results, mathematical models with regression equations for the surface roughness parameters Ra and Rz were developed. The ANOVA analysis was used to perform the results analysis. Table 4 and Table 5 present the results of ANOVA variance analysis. The impact of the cutting data (*f* and *v_c_*) on the surface roughness parameters is shown in Figure 7 and Figure 8.

Due to the strongly nonlinear impact of the tilted angle of the surface, the regression Equations (1) and (2) were determined for two domains, such as: *δ* ≤ −8° and *δ* ≥ −8°.

(1) $$Ra=\{\begin{array}{c} 1.053+1.371\cdot f+0.0705\cdot \delta +0.001179\cdot \delta^{2}+0.001524\;\cdot v_{c},\;\;\;\;\;\;\;\;for\;\;\delta \leq -8{}^{\circ}\\ 0.3909+2.052\cdot f\;-\;0.02138\cdot \delta +0.001018\cdot \delta^{2}+0.000117\;\cdot v_{c},\;\;for\;\;\delta >-8{}^{\circ} \end{array}$$

(2) $$Rz=\{\begin{array}{l} 7.668+5.06\cdot f+0.578\cdot \delta \;+0.01519\cdot \delta^{2}-0.00126\;\cdot v_{c},\;\;\;\;\;\;\;\;\;for\;\;\delta \leq -8{}^{\circ}\\ 2.002+11.19\cdot f-0.09332\cdot \delta +0.007577\cdot \delta^{2}+0.00069\;\cdot v_{c},\;\;for\;\;\delta \geq -8{}^{\circ} \end{array}$$

The ANOVA variance analysis results indicate the lack of effect of the cutting speed on the surface roughness parameters (*p* = 0.531 for Ra and *p* = 0.355 for Rz). As a result, the regression equations can be expressed as follows:

(3) $$Ra=\{\begin{array}{l} 1.170+1.371\cdot f+0.0715\cdot \delta +0.00131\cdot \delta^{2},\;\;\;\;\;\;\;\;for\;\;\delta \leq -8{}^{\circ}\\ 0.412+2.052\cdot f\;-\;0.0210\cdot \delta +0.00092\cdot \delta^{2},\;\;\;\;\;\;\;for\;\;\delta \geq -8{}^{\circ} \end{array}$$

(4) $$Rz=\{\begin{array}{l} 8.397+5.064\cdot f+0.7321\cdot \delta \;+0.02220\cdot \delta^{2},\;\;\;\;\;\;\;\;for\;\;\delta \leq -8{}^{\circ}\\ 2.195+11.191\cdot f-0.0859\cdot \delta +0.00655\cdot \delta^{2},\;\;\;\;\;\;\;\;for\;\;\delta \geq -8{}^{\circ} \end{array}$$

**Figure 9:**
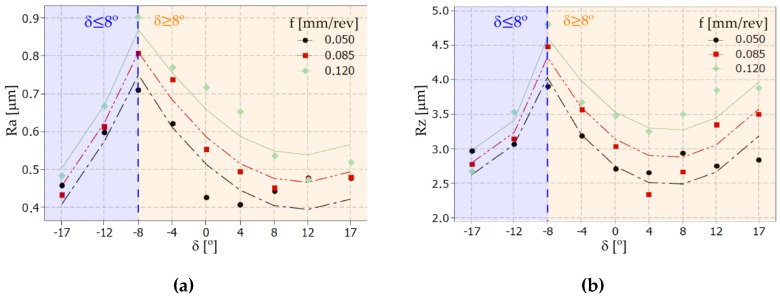
Graphs representing the relation between the tilted angle *δ* of the machined surface and the surface roughness parameters: (**a**) Ra and (**b**) Rz*;* for different feed rate parameters values.

For the above reasons, the mathematical model and prediction method focus on the impact of feed and tilted angle of surface on the values of surface roughness (Ra and Rz). In Figure 9, the calculated and measured surface roughness parameters Ra and Rz for different tilted angles of surface and feed rate values are presented.

**Figure 10:**
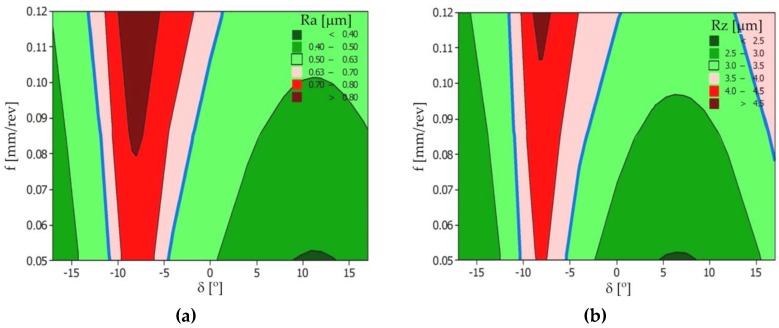
Graphs representing the relation between the tilted angle *δ* of the machined surface and the feed rate *f* for the surface roughness parameters: (**a**) Ra; and (**b**) Rz.

The contour maps were created based on the measured spherical surface roughness (Ra and Rz) for selected tilted surface angles *δ*. The area of possible solutions determined by using the mathematical models is depicted in Figure 10. The zones with red color include the IT7 class of the surface roughness (Ra = 1.25 μm, Rz = 6.3 μm). However, the zones with green color include the IT8 class (Ra = 0.63 μm, Rz = 3.2 μm).

### 2.3. Designed Feed Rate Selection Method

**Figure 11:**
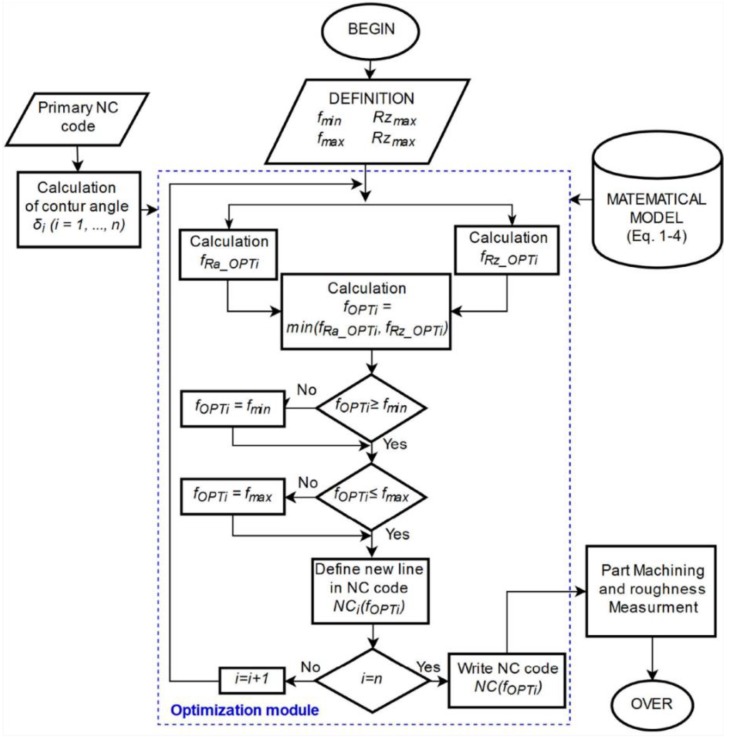
Algorithm of the optimization method of feed rate values.

This section presents a method of selecting a feed rate that enables the machining of a curved surface, characterized by the acceptable and aligned values of the roughness surface parameters (Ra, Rz), regardless of the tilted surface angle. In the first step, a mathematical model is constructed describing the influence of the cutting speed and feed rate on the values of the roughness parameters of the surface fragments inclined at different angles *δ*. Initial parameters, such as Ra_max_, Rz_max_*, f_min_,* and *f_max_*, are also determined and transferred to an optimization module. In the second step, the NC machining code is analyzed. On the basis of the results analysis, a machined surface contour and values of the surface tilted angles *δ_I_ (I = 1…n)* are defined. In the optimization module, the values of the feed rates *f_Ra_OPTi_* and *f_Rz_OPTi_* are calculated based on the mathematical model equations (Equations (1)–(4)) and tilted angle values *δ_i_*. In the next step of the optimization procedure realization, the feed rate *f_OPTi_* is defined as lower values of the feed rates *f_Ra_OPTi_* and *f_Rz_OPTi_*_­_. Then, the inequalities, such as *f_OPTi_ ≥ f_min_* and *f_OPTi_ ≤ f_max_,* are checked. If both inequalities are true, the value of the feed rate *f*_OPTi_ is applied to the NC code *NC_i_ (f_OPTi_)*. If one of them is not true, the value of the feed rate *f_OPT_* is replaced by *f_min_* or *f_max_* (this depends on the unfulfilled criterion) and applied to the NC code *NC_i_ (f_OPTi_)*. Then, the correctness of the NC code is investigated by performing all iterations (*i = n*). If the equality is not true, the next iteration is performed (*i = i* + 1). If the criterion is fulfilled, the NC code is generated as the optimized NC code and applied to create the prototype of the part. At the end of the optimization procedure, surface roughness measurements (Ra and Rz) are performed to verify the correctness of the obtained optimization results. The developed method is presented in Figure 11.

## 3. Case Study—Method Verification

**Figure 12:**
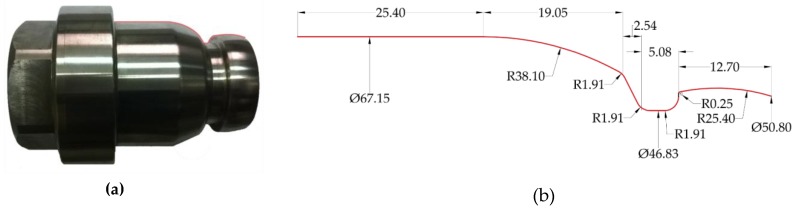
(**a**) The component; (**b**) analyzed surface of the part and its geometrical dimensions.

The verification of the proposed method was performed based on an experimental test. During the experiment, the surface of a Ti6-Al-4V alloy part, marked by the red line in Figure 12, was machined.

The selected surface was machined by using the cutting insert with symbol N123F1-0318-RO S05F. In the first part of the research, the following cutting data with constant values was applied: depth of cut *a_p_* = 0.7 mm, feed rate *f* = 0.085 mm/rev and cutting speed *v_c_* = 80 m/min. The surface was characterized by the required surface roughness class IT8 (Ra = 0.63 μm, Rz = 3.2 μm). On the machined surface, changes in the surface roughness values were observed. The changes were analogous to those described in Section 2.1 and Section 2.2.

**Figure 13:**
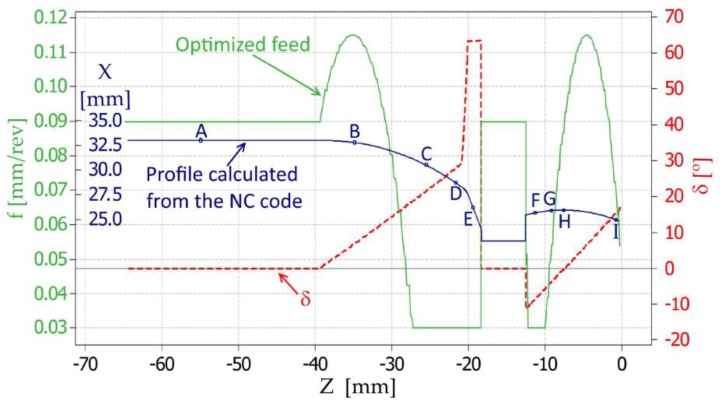
Graph representing the calculated feed rate values *f_OPTi_* and the marked contour of the examined surface.

The second part of the research consisted of generating and optimizing the NC code according to the algorithm presented in Figure 11. Due to the possibility of saving only one feed rate in the one code line (one tool movement), the analyzed contour of the part was divided into smaller fragments with a length 0.5 mm. Next, the tilted angle *δ_I_* of the contour and the initial parameters (such as: *f_min_* = 0.03 mm/rev, *f_max_* = 0.14 mm/rev, Ra_max_ = 0.63 μm and Rz_max_ = 3.2 μm) were determined. In the next step, feed rate optimization was performed on the basis of the mathematical model, presented in the Section 2.2 (Equations (3) and (4)). The calculated feed rate values (*f_Ra_OPTi_*, *f_Rz_OPTi_* and *f_OPTi_)* were compared with the limit values *f_min_* and *f_max_*. In Figure 13, the optimized feed rate values *f_OPT_*_­_ and the marked contour of the machined surface are presented. 

**Figure 14:**
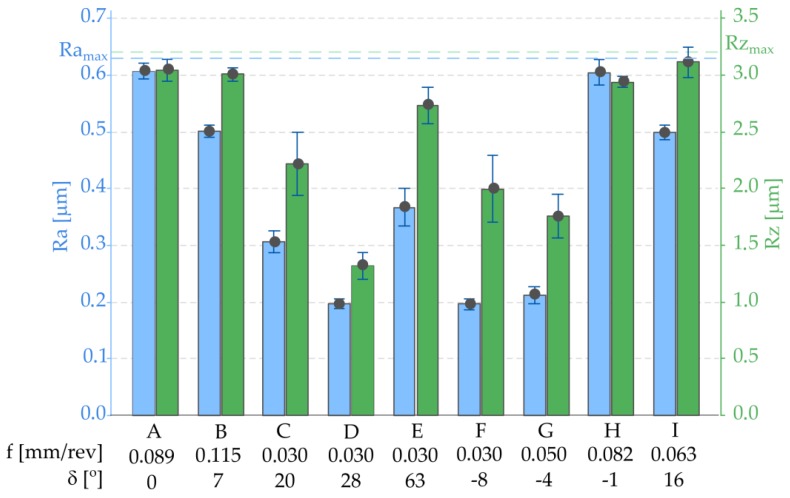
Graph representing the average values of the surface roughness parameters Ra and Rz in points A–I (according to Figure 13).

As a result of the feed rate optimization, the desired goal was achieved, meaning that the values of the surface roughness parameters were below their upper limit values (Ra ≤ 0.63 μm and Rz ≤ 3.2 μm) regardless of the surface shape and its tilted angle. The average values with standard deviations of the surface roughness parameters Ra and Rz, measured in points such as A–I (according to Figure 13), are shown in Figure 14.

**Figure 15:**
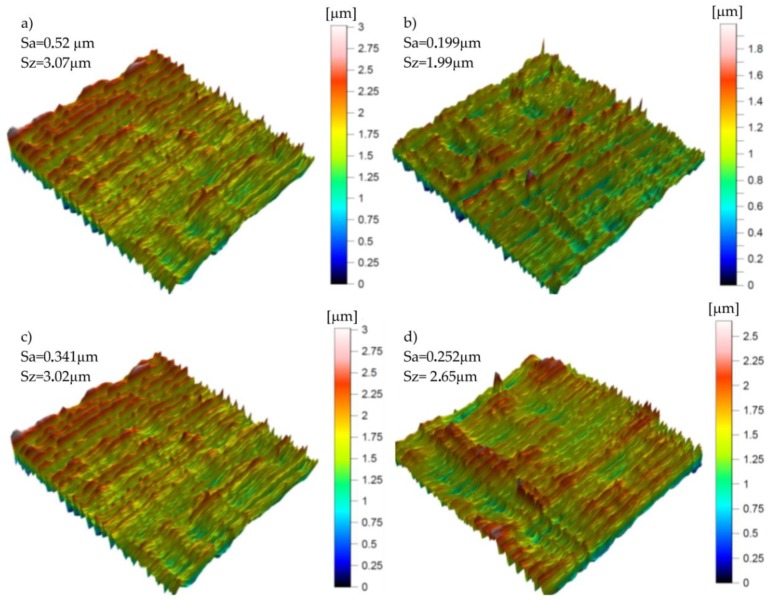
Graphs representing the isometric view of the surface topography in the points: (**a**) B; (**b**) D; (**c**) E; (**d**) G.

During the experimental tests, the measurements of 3D surface roughness were carried out. Examples of the topographies and isometric views of points such as B, D, E, and G (according to Figure 13), are shown in Figure 15.

## 4. Discussion

The results analysis of the experiment showed the significant effect of the feed rate *f* and the tilted angle of surface *δ* on the surface roughness. The results analysis indicated the insignificant impact of cutting speed on the surface roughness parameters (Ra and Rz). Similar results have been presented in the scientific literature. The optimization procedure focuses on optimizing values of the feed rate in order to obtain sufficient surface roughness, regardless of the tilted angle surface. The proposed method was examined during the case study verification. As a result of the feed rate optimization, the desired aim was achieved. Mean values of the surface roughness parameters were below their upper limit values (*Ra* ≤ 0.63 μm and *Rz* ≤ 3.2 μm) and values of feed rate were set up in a range of 0.03–0.115 mm/rev.

The optimization method proposed in this paper relies on the feed rate value modification, which affects the processing time *t_m_*. The cutting time with optimized feed rate *f_OPT_* (generated by NC code) is 160 s. The machining time when applying a constant feed rate *f* = 0.085 mm/rev is 120 s. However, this value of the feed rate results in acceptable surface roughness being obtained for the curvilinear surface with the tilted angle only in the range −1° < *δ* < 12° and a flat surface with the tilted angle *δ* = 0°. The applied constant value of the feed rate *f* = 0.05 mm/rev results in a machining time of *t_m_* = 204 s and an acceptable surface roughness for the tilted angle in the range −17° < *δ* < −12° and −4° < *δ* < 17°. The acceptable surface roughness obtained for the full range of the tilted angle *δ* is available only for the constant feed rate value *f_min_* = 0.03 mm/rev, but in that case, the machining time is longer, at *t_m_* = 304 s. Thus, it causes a more than twofold increase in the cutting time in relation to the application of the optimized feed rate. 

## 5. Conclusions

The research analysis presented in this paper concerns the significant problem of the locally variable roughness of curvilinear surfaces occurring after turning. This problem appears to be important from the point of view of the quality of the surface of manufactured machine parts. The authors of the paper have developed a mathematical model for predicting the values of the curvilinear surface roughness parameters Ra and Rz. The method for optimization of cutting data (cutting speed and feed rate) for machining the curvilinear surfaces was proposed by taking into account the alignment of the surface roughness parameters. The research indicated the insignificant effect of the cutting speed on the surface roughness parameters. This means that the cutting speed value has been correctly selected in the investigations, meaning that in this case, the regression equation can be simplified to optimize only the feed rate and tilted angle. 

The case study presented in the second part of the paper verified the correctness of the developed machining strategy. According to the proposed optimization method, the machining time of the example curvilinear surface of the titanium alloy workpiece was shortened by almost half in comparison to the non-optimized cutting process for the full range of the tilted angle *δ*. Surface roughness parameters were below their upper limit values (Ra ≤ 0.63 μm and Rz ≤ 3.2 μm) regardless of the surface shape and its tilted angle.

In the literature, curvilinear surfaces are more often machined using a milling process, so the proposed optimization method can increase the applicability of the turning process to create the curvilinear surfaces with acceptable and aligned surface roughness parameters.

In the future, the authors plan a modification of the proposed method to apply the new calculation method based on a neural network. The approach should make it possible to provide a more accurate prediction of surface roughness parameters and the impact of additional cutting data such as cutting depth, radius of surface curvature, tool wear and total cutting force components.

## Figures and Tables

**Table 1:** Chemical composition of Ti6Al4V (wt. %).

Ti	Al	V	Fe	C	N	O	Y	H
Balance	6.3	4.1	0.03	0.01	0.01	0.16	<0.005	<0.002

**Table 2:** Mechanical properties of Ti6Al4V.

Tensile Strength (MPa)	Yield Strength 0.2 % (MPa)	Elongation (%)	Reduction of Area (%)
1000	900	15	41

**Table 3:** Experimental design of variables.

**Surface No.**	1	2	3	4	5	6
***a_p_* [mm]**	0.7 mm					
***f* [mm/rev]**	0.05	0.085	0.12	0.085	0.085	0.085
***v_c_* [m/min]**	80	80	80	60	70	90

**Table 4:** Analysis of ANOVA variance for surface roughness parameter Ra.

Source	DF	Adj SS	Adj MS	F-Value	P-Value
*v_c_*	3	0.00647	0.002156	0.74	0.531
*f*	2	0.20578	0.102890	35.18	0.000
*δ*	8	2.18288	0.272861	93.29	0.000
Residual Error	148	0.43288	0.002925	-	-
Total	161	2.83945	-	-	-

**Table 5:** Analysis of ANOVA variance for surface roughness parameter Rz.

Source	DF	Adj SS	Adj MS	F-Value	P-Value
*v_c_*	3	0.3054	0.10179	1.09	0.355
*f*	2	5.5346	2.76728	29.64	0.000
*δ*	8	45.4030	5.67538	60.80	0.000
Residual Error	148	13.8161	0.09335	-	-
Total	161	66.1300	-	-	-

## References

[1] Ravi Kumar S.M., Kulkarni S.K. (2017). Analysis of Hard Machining of Titanium Alloy by Taguchi Method. Mater. Today Proc..

[2] Venkata Ramana M., Shanmuka Aditya Y. (2017). Optimization and Influence of Process Parameters on Surface Roughness in Turning of Titanium Alloy. Mater. Today Proc..

[3] Sun J., Huang S., Wang T., Chen W. (2018). Research on surface integrity of turning titanium alloy TB6. Procedia CIRP.

[4] Ramesh S., Karunamoorthy L., Palanikumar K. (2012). Measurement and Analysis of Surface Roughness in Turning of Aerospace Titanium Alloy (gr5). Measurement.

[5] Ezugwu E.O., Wang Z.M. (1997). Titanium alloys and their machinability—A review. J. Mater. Process. Technol..

[6] Ślusarczyk Ł., Struzikiewicz G. (2014). Hardened steel turning by means of modern CBN cutting tools. Key Eng. Mater..

[7] Kowalczyk R., Matras A., Zębala W. (2014). Analysis of the surface roughness after the sintered carbides turning with PCD tools. Proc. SPIE-Int. Soc. Opt. Eng..

[8] Yusup N., Zain A.M., Hashim S.Z.M. (2012). Evolutionary techniques in optimizing machining parameters: Review and recent applications (2007–2011). Expert Syst. Appl..

[9] Rao R.V., Rai D.P., Balic J. (2017). A multi-objective algorithm for optimization of modern machining processes. Eng. Appl. Artif. Intell..

[10] Yang J., Wang X., Kang M. (2018). Finite Element Simulation of Surface Roughness in Diamond Turning of Spherical Surfaces. J. Manuf. Process..

[11] Yang A., Han Y., Pan Y., Xing H., Li J. (2017). Optimum Surface Roughness Prediction for Titanium Alloy by Adopting Response Surface Methodology. Results Phys..

[12] Qehaja N., Jakupi K., Bunjaku A., Bruçi M., Osmani H. (2015). Effect of Machining Parameters and Machining Time on Surface Roughness in Dry Turning Process. Procedia Eng..

[13] Kumar Khare S., Agarwal S., Srivastava S. (2018). Analysis of Surface Roughness during Turning Operation by Taguchi Method. Mater. Today Proc..

[14] Zȩbala W., Gawlik J., Matras A., Struzikiewicz G., Ślusarczyk Ł. (2014). Research of surface finish during titanium alloy turning. Key Eng. Mater..

[15] D’Mello G., Pai S. (2018). Surface Roughness Modeling in High Speed Turning of Ti-6Al-4V Using Response Surface Methodology. Mater. Today Proc..

[16] Asiltürk I., Akkus H. (2011). Determining the Effect of Cutting Parameters on Surface Roughness in Hard Turning Using the Taguchi Method. Measurement.

[17] Makadia A.J., Nanavati J.I. (2013). Optimisation of machining parameters for turning operations based on response surface methodology. Measurement.

[18] Yamane Y., Ryutaro T., Tadanori S., Ramirez I.M., Keiji Y. (2017). A new quantitative evaluation for characteristic of surface roughness in turning. Precis. Eng..

[19] Wang X., Feng C.X. (2002). Development of Empirical Models for Surface Roughness Prediction in Finish Turning. Int. J. Adv. Manuf. Technol..

[20] Özel T., Karpat Y. (2005). Predictive modeling of surface roughness and tool wear in hard turning using regression and neural networks. Int. J. Mach. Tools Manuf..

[21] Beatrice B.A., Kirubakaran E., Thangaiah P.R.J., Wins K.L.D. (2014). Surface Roughness Prediction using Artificial Neural Network in Hard Turning of AISI H13 Steel with Minimal Cutting Fluid Application. Procedia Eng..

[22] Vyboishchik A.V. (2016). Modelling Topology of Freeform Surfaces with Ball-end Milling. Procedia Eng..

[23] Bey M., Cherfi A. (2018). Finishing of freeform surfaces with an optimized Z-Constant machining strategy. Procedia CIRP.

[24] Bouzakis K.-D., Aichouh P., Efstathiou K. (2003). Determination of the chip geometry, cutting force and roughness in free form surfaces finishing milling, with ball end tools. Int. J. Mach. Tools Manuf..

[25] Antoniadis A., Savakis C., Bilalis N., Balouktsis A. (2003). Prediction of Surface Topomorphy and Roughness in Ball-End Milling. Int. J. Adv. Manuf. Technol..

[26] Zhou X., Zuo C., Liu Q., Lin J. (2016). Surface generation of freeform surfaces in diamond turning by applying double-frequency elliptical vibration cutting. Int. J. Mach. Tools Manuf..

